# Immune Thrombocytopenia in a Patient Recovering From COVID-19

**DOI:** 10.1097/HS9.0000000000000419

**Published:** 2020-09-30

**Authors:** Symeon Metallidis, Georgia Gioula, Maria Papaioannou, Maria Exindari, Theofilos Chrysanthidis, Olga Tsachouridou, Iordanis Mimtsoudis, Maria Christoforidi, Pantelis Zempekakis, Anna Papa-Konidari, Sotirios Tsiodras

**Affiliations:** 1 Infectious Diseases Unit, 1st Internal Medicine Department, AHEPA University Hospital, Thessaloniki, Greece; 2 Department of Microbiology, Medical School, Aristotle University of Thessaloniki, Greece; 3 4th Academic Department of Medicine, National and Kapodistrian University of Athens, Medical School, Athens, Greece.

COVID-19 has prominent hematologic manifestations and is often correlated with significant blood deficiencies like lymphocytopenia, thrombocytopenia, and hypercoagulopathy.^1^ These defects are mainly present in severe COVID-19 cases.^2^ Precise evaluation of baseline laboratory indices and over the course of the disease assist clinicians to improve management and therapeutic interventions to these patients and to recognize promptly lethal complications, leading to better outcomes.

A 33-year-old woman with diabetes mellitus type 1 under insulin pump, and a 2-day history of mild myalgias, pharyngalgia, and low-grade fever presented to the emergency department. No cough or shortness of breath was reported. She confirmed recent contact exposure twice to the first confirmed COVID-19 case in Greece.

The initial physical examination revealed a body temperature of 37.3^o^C, a pulse of 94 beats per minute, respiratory rate of 16 breaths per minute, oxygen saturation of 98% on ambient air. Lung auscultation was normal and baseline chest radiography revealed no pathologic findings from the thorax. Baseline complete blood count showed a normal leukocyte count (7.41 × 10^3^/μL) with mild lymphopenia (1.1 × 10^3^/μL), hemoglobin level (14.1 g/dL) and a normal platelet count (165 × 10^3^/uL).

The C-reactive protein level, ferritin, lactate dehydrogenase, liver-function tests, and fibrinogen were within normal ranges. A polymerase chain reaction (PCR) test was performed for Influenza type A and B which was negative. An oropharyngeal swab specimen was also obtained to test for SARS-CoV-2. A real-time RT-PCR targeting the envelope (E) gene and the RNA-dependent RNA polymerase (RdRp) gene was applied, using the Viral RNA mini kit (Qiagen, Hilden, Germany),^3^ which was reported back as positive within 4 hours.

Upon the confirmation of diagnosis and the outbreak of SARS-CoV-2 in Greece on February 26, 2020, the patient was admitted in airborne-isolation ward for further investigation and follow-up despite her mild clinical condition. No drugs or heparin were administered and the patient received only hydrating supportive care at admission.

**Figure 1:**
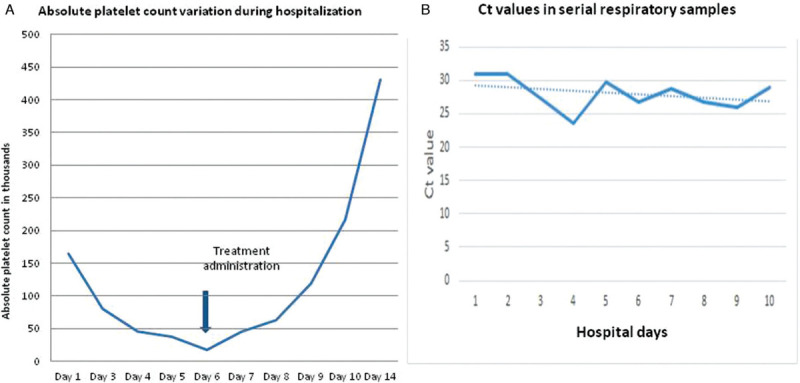
A. Absolute platelet count variation during hospitalization. B. Cycle threshold values in serial upper tract respiratory samples of case during hospitalization.

On day 3 (day 5 of illness), symptoms were fully resolved. Laboratory tests remained normal, while viral-RNA remained detectable in serial oropharyngeal specimens during hospitalization (Fig. 1).

Two days later while she remained asymptomatic, isolated thrombocytopenia (platelet count 37 × 10^3^/uL) was noted. Blood sampling was repeated to confirm the drop in platelets. A thorough clinical examination was then performed. The patient had a normal Glasgow Coma Scale score, no signs of jaundice were present, liver and spleen were not palpable and there was no evidence of any hemorrhagic manifestation or hemodynamic instability.

On day 6, platelets further dropped to 18 × 10^9^/L. Evaluation of a peripheral blood smear excluded pseudothrombocytopenia or other hematological conditions. Further virological laboratory tests for hepatitis B surface antigen (HBsAg) and HBV anti-core antibodies, hepatitis C, anti-HAV IgG, Epstein–Barr virus (EBV) antibodies, cytomegalovirus antibodies (CMV), and HIV Elisa (fourth generation), which all evidenced negative for current infection. Quantitative serum immunoglobulin levels (IgG, IgA, IgE, and IgM) and complement levels were normal. The partial thromboplastin time (PTT), fibrinogen level, and D-dimer level were also normal. Direct Coombs test, antinuclear and antiphospholipid antibodies were negative.

Serum immunoglobulin levels, complement levels, partial thromboplastin time, fibrinogen and d-dimers were within reference ranges. The exclusion diagnosis of immune thrombocytopenia was made. Bone marrow examination was not considered, due to isolated thrombocytopenia with normal features on physical examination and blood smear.^4,5^

A combination of a short course of dexamethasone and intravenous immunoglobulin (IVIg) was initiated. Thus, 24 mg of dexamethasone per day for 4 days and 1 g/kg/day of IVIG for 2 consecutive days were administered. Platelet count recovered adequately during hospitalization (Fig. 1).

Many studies suggest an association between viral infections and ITP onset.^6^ CMV and herpes virus has been associated with refractory immune thrombocytopenia.^7,8^ EBV has been associated with anti-platelet antibodies formation,^3^ implying the interference of chronic viral infections in ITP onset.

Acute viral infections and immunization have been associated with usually transient thrombocytopenia. Some acute or persistent infections might lead to perpetuate thrombocytopenia.^9^ However, the role of acute viral infections remains unknown in ITP induction. A few cases of flavivirus infections have been reported exacerbating chronic ITP or provoking first onset, like Dengue and more recently Zika, representing a challenge for clinicians.^10,11^

Recently, severe complicated immune thrombocytopenia was reported in a patient with coronavirus (CoV) infection.^12^ Hematological alterations in patients with severe acute respiratory syndrome (SARS) caused by CoV, may include thrombocytopenia caused by different mechanisms. SARS-CoV may trigger autoimmune antibody or immune complexes formation, but also can directly affect megakaryocytes, hematopoietic stem cells and platelets.^13^ Very recently, a unique case of Immune Thrombocytopenic Purpura (ITP) was reported as a primary finding in a severely ill SARS-CoV-2 infected adult, implying a correlation of the virus and ITP.^14^

Detection of SARS-CoV-2 RNA in oropharyngeal specimens from the upper respiratory tract with low cycle threshold (Ct) values on hospital day 4 (Fig. 1) is suggestive of high viral load and potential for transmission, but provides no information on the clinical impact on the patient according to published data.^15^ Furthermore, literature is still inconclusive on the time that the RNA of the virus becomes undetectable in respiratory secretions and varies depending on the severity of the disease and the linkage with complications of the disease.^15^

The transient course in this case speculates, but does not confirm a causal relation between COVID-19 and immune thrombocytopenia.^2,14^ Since our understanding continues to evolve, the spectrum of clinical and hematological manifestations characterizing this emerging infection will be further elucidated in the forthcoming months. At the time of submission, to the best of our knowledge this is the second case of COVID-19 induced immune thrombocytopenia in the literature. However, our patient presented mild disease and thrombocytopenia occurred post recovery of the symptoms. This report highlights the need for vigilant monitoring for complications associated with COVID-19, even in asymptomatic patients.
